# Psychosocial and other working conditions in relation to body mass index in a representative sample of Australian workers

**DOI:** 10.1186/1471-2458-6-53

**Published:** 2006-03-02

**Authors:** Aleck S Ostry, Samia Radi, Amber M Louie, Anthony D LaMontagne

**Affiliations:** 1 Department of Health Care and Epidemiology, University of British Columbia, 5804 Fairview Avenue, Vancouver BC, V6T 1Z3, Canada; 2 Department of Epidemiology and Preventive Medicine, Monash University, Melbourne, Australia; 3 Unité INSERM 558, Toulouse, France; 4 Centre for Health & Society, School of Population Health, University of Melbourne, 207 Bouverie Street, Melbourne VIC 3070, Australia

## Abstract

**Background:**

The aim of the study was to examine the relationship between psychosocial and other working conditions and body-mass index (BMI) in a working population. This study contributes to the approximately dozen investigations of job stress, which have demonstrated mixed positive and negative results in relation to obesity, overweight and BMI.

**Methods:**

A cross-sectional population-based survey was conducted among working Australians in the state of Victoria. Participants were contacted by telephone from a random sample of phone book listings. Information on body mass index was self-reported as were psychosocial work conditions assessed using the demand/control and effort/reward imbalance models. Other working conditions measured included working hours, shift work, and physical demand. Separate linear regression analyses were undertaken for males and females, with adjustment for potential confounders.

**Results:**

A total of 1101 interviews (526 men and 575 women) were completed. Multivariate models (adjusted for socio-demographics) demonstrated no associations between job strain, as measured using the demand/control model, or ERI using the effort/reward imbalance model (after further adjustment for over commitment) and BMI among men and women. Multivariate models demonstrated a negative association between low reward and BMI among women. Among men, multivariate models demonstrated positive associations between high effort, high psychological demand, long working hours and BMI and a negative association between high physical demand and BMI. After controlling for the effort/reward imbalance or the demand/control model, the association between physical demand and working longer hours and BMI remained.

**Conclusion:**

Among men and women the were differing patterns of both exposures to psychosocial working conditions and associations with BMI. Among men, working long hours was positively associated with higher BMI and this association was partly independent of job stress. Among men physical demand was negatively associated with BMI and this association was independent of job stress.

## Background

The prevalence of overweight and obesity has been high in most industrialized nations, since the early 1950s; however, this trend accelerated in the 1990s. Australia has not escaped this phenomenon [1]. The risk of cardiovascular disease increases with overweight and obesity [2]. According to the Australian National Health Survey conducted in 2001, 16% of men and 17% of women (aged more than 18) were obese, 42% of men and 25% of women were overweight, and 1% of men and 5% of women were underweight [1].

An extensive literature has been published on job stress and cardiovascular disease (CVD), mainly among men [5]. This literature demonstrates that job strain and high effort/low reward conditions predict CVD [6-8], but the relative contributions of direct and indirect mechanisms remain unclear. Some evidence suggests indirect effects of psychosocial and other work conditions on health through health behaviours [5].

Indirect pathways may include effects of job stress on physical activity, eating behaviours, and other behaviours that may be related to BMI [9]. Previous studies have found associations between working conditions and health behaviours, such as diet, physical activity and alcohol consumption, which impact weight change [10-12]. For example, in a cross-sectional analysis of Japanese workers (n = 6,759) job strain was associated with low vegetable and high alcohol consumption [11]. Another cross-sectional survey of American workers (n = 3,843) showed that job demands were positively associated with high fat intake in men, while decision latitude was positively associated with physical activity in both men and women [12]. More recently, the Helsinki Health Study (n = 6,243) found that among women, mentally strenuous work and high job control were associated with a healthy diet [10].

The two most widely used instruments to measure occupational stress are Karasek's demand/control (DC) and Siegrist's effort/reward imbalance (ERI) models. The demand/control model focuses on task-level job characteristics. It postulates that psychological strain results from the interaction of job demands and job control, with the combination of low control and high demands producing "job strain". In contrast, the effort/reward imbalance model includes personal characteristics of the worker (i.e., over commitment) and also conceptualizes and measures work conditions more broadly than the demand/control model. It focuses on the reciprocity of exchange at work where high costs/low gain conditions (i.e., high effort and low reward) are considered particularly stressful.

While the demand/control and effort/reward imbalance models have been tested in relation to CVD outcomes they have been less widely investigated in relation to risk factors for CVD including body mass index (BMI) [12]. Nonetheless, fourteen studies have been conducted using job stress to test for associations with body mass index [4,13-25]. The DC model was used in ten of these studies [12-16,20,21,23-25], two of which also used the ERI model [13,14].

Six of the studies with the DC model showed a positive and statistically significant relationship with BMI [12-15,20,24] but the remaining four showed no association. Both studies with the ERI model showed a positive and statistically significant relationship with BMI [13,14]. And, two of the remaining four studies utilizing other measures of job strain showed positive and statistically significant relationships with BMI [4,18].

In these positive investigations with the DC and ERI models, high job strain [13], low control [13,24] and high ERI [13] have been linked with increased BMI. Among the four workplace studies of stress and obesity that did not use the DC or ERI models, the results varied [4,22,26,27]. House et al. [22], using data from the Tecumseh Community Health Study from 1967 to 1969, demonstrated negative associations between occupational position and "pressures on the job" in relation to obesity for both men and women.

In a study of 1,137 Swedish women, Rosmond and Bjorntop [4] found that education, satisfaction with management, attempts to alter work situation" (i.e., a proxy measure of participation and control), and physical exercise were all negatively associated with BMI.

In a cohort study, Kornitzer and Kittel [26] found no association between job stress and obesity. The stress measure they used in this study of obesity was also tested in relation to coronary heart disease and was also non-predictive. Conversely, Georges et al. [27] found a borderline significant positive association (P= 0.06) between job demands and increased BMI. This survey also demonstrated that men with high job strain were more likely to have a pattern of central body fat distribution then men with low job strain [27].

Non-psychosocial working conditions have been investigated in relation to body mass index also. Using data from the National Population Health Survey in Canada, Shields [28] demonstrated that, after statistical adjustment, men who worked more than 35 hours a week had an odds ratio of 1.4 for being overweight (BMI> 25). No association between long hours of work and overweight was demonstrated for women.

Using a representative survey of workers in the Australian state of Victoria, we have assessed associations of job stress (i.e., demand/control; effort-reward imbalance models), shift work, physical demand and hours worked, with BMI among men and women.

## Methods

This study was reviewed and approved by the University of Melbourne's Human Research Ethics Committee (HREC protocol #030398).

### Study design and sample

A cross-sectional population-based survey was conducted by telephone from a random sample of White Pages listings in the state of Victoria in Australia. In order to reflect general population occupational group proportions, quotas were set to match Australian Bureau of Statistics (ABS) census proportions of upper white-collar, lower white-collar, and blue-collar groups (29%, 30%, and 41%, respectively). We also quota sampled for urban/Melbourne (72%) versus rural/regional Victoria (28%). The inclusion criteria were 1) being aged 18 years or older, and 2) working at the time of the survey for profit or pay (including self-employed). Interviews were completed in November 2003 with a 66 % response rate from in-frame households (i.e., had one or more working residents aged 18 or over) to yield a representative sample of 1,101 working Victorians (526 men and 575 women.).

### Measures

A brief version [29] of Karasek's DC model [30] was used to measure psychological demand (sum of 3 items, Cronbach's alpha = 0.66) and job control (2 equally weighted scales of 6 and 3 items measuring skill discretion and decision authority respectively, Cronbach's alpha = 0.80), and physical demand at work (sum of 2 items). Each of the 3 dimensions was dichotomised at the median. Dichotomised psychological demand and job control were combined to create four categories: low strain (low demand and high control), active jobs (high demand and high control), passive jobs (low demand and low control), and job strain (high demand and low control). In subjects with missing data, scores were recalculated using the lower and the higher theoretical score for each missing item and dimensions dichotomised according to their median. If the classification of participants was the same for any possible value of the missing item, participants were considered as having non-missing answers for the dimension of interest (38/88 participants with missing data). If the classification differed according to the replaced value, participants were considered as having a missing answer for the dimension.

Siegrist's ERI model [8] was used to measure effort (5 items, Cronbach's alpha = 0.80), reward (11 items, Cronbach's alpha = 0.81), and over commitment (6 items, Cronbach's alpha = 0.82). Effort and reward items were summed into scales. The effort and reward scales were dichotomized at the median to create two variables measuring high and low effort and high and low reward. As well, a ratio of effort to reward was computed using a correction factor to give equal weight to both scales. The ratio was dichotomised using a cut-point of 1. A ratio greater than 1 indicated that effort was higher than reward. For participants who did not answer each question in the scale, scores were calculated if at least 80% of the items were answered (4/5 items for effort and 9/11 items for reward) [32]. Participants exposed to over commitment were defined as those in the sample upper tertile.

Shift work was defined as work performed at least partly during the night, excluding day shift work and those who worked exclusively during the day. Weekly working hours were calculated as the average number of hours worked per week over the previous month, and treated categorically as up to Australian standard full-time hours (≤ 35 hours/week), long working hours (36–49 hours/week), and excessive working hours (≥ 50 hours/week).

Demographic and other covariate data were collected on age (treated categorically as < 30 years, 30–40 years, 41–50 years, and 51+ years), highest level of education completed (post-graduate, undergraduate, vocational, high school completion, and some primary or secondary school completion), and children living at home. Occupations were collapsed into three categories (upper white, lower white, and blue-collar). Marital status was assessed as living as a couple versus living alone. Hostility was assessed using the sum of a 3-item 5-point Likert scale [33] with higher scores indicating greater hostility. This was dichotomized at the median.

### Outcomes

Body mass index was based on self-reports, calculated as weight in kilograms divided by height in meters squared. Information on BMI was missing for 7 men and 43 women.

### Statistical analysis

Linear regression analyses were conducted with the SPSS statistical package (version 12, SPSS Inc., Chicago, 2003) to assess the relationship of BMI with socio-demographics and working conditions. Categorical variables with more than two categories were included in models as dummy variables. All analyses were conducted separately for males and females. One observation was excluded from analyses: a woman with BMI = 63.7. This was a marked outlier that would be likely to unduly influence regression analyses because the nearest observation in the distribution was a BMI of 44.6. Descriptive statistics and mean value for BMI by socio-demographics and working conditions were first calculated for women and men. All multivariate models controlled for age, education, and marital status.

Finally, job strain and effort-reward imbalance were modeled alone and in combination to assess the independence of observed relationships between working conditions and BMI, and to comprehensively assess the full range of measured psychosocial working conditions in relation to BMI with simultaneous adjustment.

## Results

Descriptive statistics and mean value for BMI by socio-demographics and psychosocial and other working conditions for women and men are shown in Table 1. Women had a lower mean BMI than men 24.5 versus 26.1 (p < 0.001). For men and women, mean BMI increased significantly with age, education level and living with a partner. For men, both high effort and working longer hours were significantly associated with a higher mean BMI. And, high physical demand was significantly associated with lower BMI. For women, low reward was associated with lower mean BMI. Hostility and living with children were not significantly related to BMI and therefore these variables were not included in the subsequent multivariate analysis.

The results of multivariate linear regression analyses for women and men are shown in Table 2. After adjusting for age, education and marital status, for men, working more than 35 hours a week, high psychological demand, and high effort were associated with increased BMI, and a negative association was observed between high physical demand and BMI. Among women, after controlling for potential confounders, a negative association was found between low reward and BMI.

We conducted a final set of multivariate analyses including statistically significant work-related predictors from bivariate analyses along with the DC and ERI models, singly and in combination, with adjustment for sociodemographic variables. Model 1 in Table 3 shows that DC measures are not significantly associated with BMI when modeled along with other work-related predictors. Models 2 and 3 show that ERI is also not associated with BMI, either independently or in combination with DC measures. The negative assocaiton between high physical demand and BMI remains stable and unaffected by inclusion of either or both job stress measures (Table 3, bottom row). Associations with longer (35–49 hours/week) and excessive (>50 hours/week) working hours are attentuated slightly by inclusion of DC and ERI job stress models singly, but when both the DC and ERI models are added to the model, working long hours becomes statistically non-significant (Model 3).

Model 4 is the most parsimonious final model presenting significant work-related predictors of BMI in men for our sample. The unstandardised regression coefficients show that men with high physical demand have a mean BMI that is 1.04 BMI units less than those in low physical demand jobs. In contrast, men with longer working hours have a mean BMI that 1.37 units higher than those working up to full time, and increasing in stepwise fashion, those working excessive hours have a mean BMI that is 1.88 units higher than those working up to full time.

For women, there were no statistically significant associations between working conditions and BMI after including DCM and ERI, independently or in combination, along with socio-demographic adjustments (data not shown).

## Discussion

There are four main results which arise from this study. First, while the effort/reward imbalance ratio itself is not associated with BMI, high effort in men and low reward in women were associated with BMI. Second, for men, high psychological demand, as measured in the DC model, was positively associated with BMI. Third, after controlling for main job stress measures (ERI or DC), for men, longer and excessive working hours were positively – and physical demand was negatively – associated with BMI. Fourth, among women, after similar adjustments neither psychosocial or other working conditions were significantly associated with BMI.

Our population-based sample is representative of occupational groups in the general population. Therefore, our findings can be generalised to the general working population in Victoria. Further, this study was strengthened by the comparative assessment of a range of psychosocial and other working conditions (two measures of job stress, working hours, physical demand, and shift work).

However, three limitations must be taken into account. First, the cross-sectional design of our study cannot support causal inferences between occupational factors and BMI. Second, information on independent and dependant variables were collected using self-reports. Even if the questionnaire was designed to minimize self-report bias in responses, some items may be subject to this type of bias. In particular, psychological demand has been shown to have a strong subjective component. And, self-reports of BMI produce under-estimates as men tend to overestimate their height and women tend to under-estimate their weight. Use of self-reports for BMI, as in this paper, will tend to attenuate any associations observed.

Studies of psychosocial working conditions and BMI have demonstrated an association between high job strain [13], low control [13,24], and high ERI [13], "pressures on the job" [22], a proxy measure of participation and control [4], and a borderline association between high psychological demand and BMI [27]. These results indicate that demands, control, or some combination of these may be associated with increased BMI but clear results from this small number, of largely cross-sectional studies, do not emerge.

The results from our study suggest that "demand" rather than "control" factors may be more salient in relation to BMI, at least for men. After fully controlling for confounding, two workload-related factors, psychological demand (from the DC model) and working long hours were both strongly associated with BMI. As well, in univariate models high effort was associated with BMI. Given that high effort combines measures of physical and psychological demand into a single variable, and given that high physical demand is negatively associated with BMI and high psychological demand with increased BMI, the effects of one may cancel the effects of the other, at least in relation to *BMI*.

There are plausible mechanisms through which long working hours could be related to BMI. Working long or excessive hours may reduce the opportunity for leisure time physical activity, might also increase the frequency of eating higher caloric value take-away or restaurant food, and might also lead to higher alcohol consumption as an unwinding or coping mechanism. It is important to note also that this assocaiton is of a similar magnitude to the effect of increasing age on men (data not shown, highest age group [51+ years)] has mean BMI 1.60 units higher than youngest [<30 years]).

In this study, while half the men worked longer working hours and one third excessive working hours less than half the women worked up to full time hours, 38% worked longer hours, and only 15% worked excessive hours (Table 1). Thus, working more than 35 hours per week is mainly a male phenomenon so that the lack of observed association between working long hours and BMI among women may be due to the small numbers of women working long hours.

## Conclusion

Our findings suggest that psychosocial work conditions may impact BMI, particularly among men, and that largely independently of job stress, both low physical demand at work and longer working hours among men may increase BMI.

## Abbreviations

BMI = Body Mass Index

CVD = Cardio-vascular Disease

ERI = Effort/reward Imbalance

DC = Demand/control

## Competing interests

The author(s) declare that they have no competing interests.

## Authors' contributions

All authors read and approved the final manuscript.

ASO:- Developed the conceptual and analytical models. Developed and wrote the literature review and drafted the paper.

SR:- Contributed to statistical analyses.

ADL:- Designed, conducted, and directed the Victorian Job Stress Survey, and contributed to statistical analyses and the writing of the paper.

AML:- Assisted in conducting the literature review and statistical analysis.

## Pre-publication history

The pre-publication history for this paper can be accessed here:



## Figures and Tables

**Table 1:** Mean Body Mass Index in Relation to Socio-demographics and Working Conditions in both Genders

	Men (N = 519)**			**Women (N = 531)**		
	BMI (SD)	N (%)	p value*	BMI (SD)	N (%)	p value*

Age			<0.001			<0.001
< 30 years	24.3 (3.2)	118 (22.7)		22.7 (4.8)	125 (23.5)	
30–40 years	26.0 (4.5)	157 (30.3)		24.7 (4.7)	150 (28.2)	
41–50 years	26.8 (3.8)	122 (23.5)		24.9 (4.4)	149 (28.1)	
? 51 years	27.2 (5.0)	122 (23.5)		25.7 (4.8)	107 (20.2)	
Educational level			0.037			0.036
Post-graduate	26.1 (4.7)	47 (9.1)		24.4 (3.8)	52 (9.8)	
Undergraduate	25.9 (3.7)	128 (24.9)		24.5 (4.8)	204 (38.6)	
Vocational	26.6 (3.9)	127 (24.7)		24.0 (4.4)	71 (13.4)	
High school	24.9 (5.1)	89 (17.3)		23.5 (4.5)	101 (19.1)	
Primary or secondary	26.7 (4.6)	123 (23.9)		25.5 (5.3)	101 (19.1)	
Marital status			<0.001			0.004
Living as a couple	25.1 (4.0)	216 (41.6)		23.8 (4.8)	230 (43.4)	
Living alone	26.8 (4.4)	303 (58.4)		25.0 (4.7)	300 (56.6)	
Living with children			0.767			0.536
No	26.1 (4.4)	294 (56.6)		24.4 (5.2)	272 (51.3)	
Yes	26.2 (4.2)	225 (43.4)		24.6 (4.3)	258 (48.7)	
Hostility			0.975			0.359
Low	26.1 (4.1)	272 (52.4)		24.7 (5.0)	243 (45.8)	
High	26.1 (4.6)	247 (47.6)		24.3 (4.6)	288 (54.2)	
Occupational category			0.531			0.156
Upper white collar	26.1 (3.9)	152 (29.3)		24.9 (4.7)	198 (37.3)	
Middle white collar	25.7 (4.8)	122 (23.5)		24.4 (4.8)	266 (50.1)	
Blue collar	26.3 (4.3)	245 (47.2)		23.6 (4.7)	67 (12.6)	
Hour worked			<0.001			0.467
<35 hours	24.2 (3.3)	77 (15.3)		24.6 (4.7)	241 (46.9)	
35 to 49 hours	26.2 (4.4)	258 (51.3)		24.2 (5.1)	198 (38.5)	
>49 hours	26.9 (4.0)	168 (33.4)		24.9 (4.5)	75 (14.6)	
Psychological work demand			0.162			0.473
Low	25.8 (3.9)	293 (59.1)		24.3 (4.9)	271 (52.0)	
High	26.3 (4.8)	203 (40.9)		24.6 (4.7)	250 (48.0)	
Control			0.144			0.119
High	26.4 (4.2)	255 (49.2)		24.9 (4.6)	214 (41.0)	
Low	25.8 (4.4)	263 (50.8)		24.2 (4.8)	308 (59.0)	
Demand/Control			0.151			0.479
Low strain	26.1 (4.3)	138 (27.9)		24.8 (4.5)	99 (19.3)	
Active jobs	26.6 (4.1)	110 (22.2)		24.8 (4.6)	112 (21.8)	
Passive jobs	25.5 (3.6)	155 (31.3)		24.0 (4.9)	167 (32.6)	
Job strain	26.1 (5.5)	92 (18.6)		24.4 (4.8)	135 (26.3)	
Effort			0.004			0.338
Low	25.6 (3.8)	290 (56.8)		24.3 (4.7)	282 (53.9)	
High	26.7 (4.9)	221 (43.2)		24.7 (4.9)	241 (46.1)	
Reward			0.938			0.024
High	26.0 (3.7)	228 (47.7)		24.9 (4.7)	261 (51.0)	
Low	26.0 (5.0)	250 (52.3)		24.0 (4.7)	251 (49.0)	
Effort/Reward ratio			0.898			0.404
? 1: Balance	26.0 (4.4)	455 (96.0)		24.5 (4.7)	488 (96.4)	
> 1: Imbalance	25.9 (4.0)	19 (4.0)		23.6 (4.1)	18 (3.6)	
Physical work demand			0.009			0.388
Low	26.7 (4.3)	202 (39.2)		24.3 (4.6)	258 (48.9)	
High	25.7 (4.3)	313 (60.8)		24.7 (4.9)	270 (51.1)	
Shift work			0.262			0.823
No	26.2 (4.3)	454 (87.5)		24.5 (4.9)	466 (87.8)	
Yes	25.5 (4.6)	65 (12.5)		24.6 (4.0)	65 (12.2)	

*Analysis of variance.

** The original sample consisted of 526 men and 575 women. BMI information was missing for 7 men so this table shows data only for (526-7) = 519 men. BMI information was missing for 43 women and one women with BMI >60 was dropped from the analysis so this table shows data only for (574-43-1) = 531 women.

**Table 2:** Conditions and Sociodemographics: Multivariate Linear Regression Analysis

	**Men (N = 427)**		**Women (N = 455)**	
	Beta*	p value	Beta	p value

High Psychological work demand (Ref = Low)	0.87	0.04	0.23	0.61
Low Control (Ref = High)	-0.40	0.34	-0.48	0.31
Demand/Control (Ref = Low strain)				
Active jobs	0.86	0.14	-0.06	0.94
Passive jobs	-0.34	0.54	-0.68	0.29
Job strain	0.50	0.42	-0.31	0.65
High Effort (Ref = Low)	1.26	>0.01	0.67	0.14
Low Reward (Ref = High)	-0.13	0.76	-0.87	0.05
Effort/Reward Ratio > 1: Imbalance (Ref = Ratio 1: Balance)**	-1.19	0.26	-0.98	0.44
Hours worked (Ref = <35 hours)				
35 to 49 hours	1.35	0.03	0.05	0.92
>49 hours	1.86	>0.01	0.48	0.47
High physical work demand (Ref=Low)	-1.15	0.01	0.30	0.50

Each model (row) adjusted for age, educational level and marital status.

* Unstandardised regression coefficient.

** Additionally adjusted for over commitment.

Ref = reference category.

**Table 3:** Body Mass Index in Relation to Working Conditions and Sociodemographics in Men: Multivariate Linear Regression Results

	**Model 1**		**Model 2**		**Model 3**		**Model 4**	
**Men (N = 427)**	Beta*	p value	Beta	p value	Beta	p value	Beta	p value

Demand/Control (Ref = Low strain)								
Active jobs	0.69	0.25	--		0.61	0.30	--	
Passive jobs	-0.14	0.80	--		-0.11	0.85	--	
Job strain	0.49	0.43	--		0.48	0.44	--	
Effort/Reward Ratio > 1: Imbalance (Ref = Ratio 1: Balance) **	--		-1.18	0.26	-1.32	0.21	--	
Hours worked (Ref = <35 hours)	1.22	0.05	1.26	0.05	1.15	0.07	1.37	0.03
35 to 49 hours	1.57	0.03	1.56	0.02	1.33	0.07	1.88	>0.01
>49 hours	-1.05	0.02	-1.05	0.02	-1.05	0.02	-1.04	0.02
High physical work demand (Ref = Low)								

Each regression model includes covariates with reported coefficients, after adjustment for age, educational level and marital status.

* Unstandardised regression coefficient.

** Additionally adjusted for over commitment.

Ref = reference category.
